# Chlorthalidone vs Hydrochlorothiazide for Hypertension Treatment After Myocardial Infarction or Stroke: A Secondary Analysis of a Randomized Clinical Trial

**DOI:** 10.1001/jamanetworkopen.2024.11081

**Published:** 2024-05-14

**Authors:** Areef Ishani, Cynthia Hau, William C. Cushman, Sarah M. Leatherman, Robert A. Lew, Peter A. Glassman, Addison A. Taylor, Ryan E. Ferguson

**Affiliations:** 1 Minneapolis VA Healthcare System, Minneapolis, Minnesota; 2 Department of Medicine, University of Minnesota, Minneapolis; 3 Cooperative Studies Program Coordinating Center, VA Boston Healthcare System, Boston, Massachusetts; 4 Medical Service, Memphis VA Medical Center, Memphis, Tennessee; 5 Department of Preventive Medicine, University of Tennessee Health Science Center, Memphis; 6 Department of Biostatistics, Boston University School of Public Health, Boston, Massachusetts; 7 Pharmacy Benefits Management Services, Department of Veterans Affairs, Washington, DC; 8 VA Greater Los Angeles Healthcare System, Los Angeles, California; 9 David Geffen School of Medicine at UCLA, Los Angeles, California; 10 Michael E. DeBakey VA Medical Center, Houston, Texas; 11 Department of Medicine, Baylor College of Medicine, Houston, Texas; 12 Department of Medicine, Boston University Chobanian & Avedisian School of Medicine, Boston, Massachusetts

## Abstract

**Importance:**

Patients with prior myocardial infarction (MI) or stroke have a greater risk of recurrent cardiovascular (CV) events.

**Objective:**

To evaluate the association of chlorthalidone (CTD) vs hydrochlorothiazide (HCTZ) with CV outcomes and noncancer deaths in participants with and without prior MI or stroke.

**Design, Setting, and Participants:**

This was a prespecified secondary analysis of the Diuretic Comparison Project (DCP), a pragmatic randomized clinical trial conducted within 72 participating Veterans Affairs health care systems from June 2016 to June 2021, in which patients aged 65 years or older with hypertension taking HCTZ at baseline were randomized to continue HCTZ or switch to CTD at pharmacologically comparable doses. This secondary analysis was performed from January 3, 2023, to February 29, 2024.

**Exposures:**

Pharmacologically comparable daily dose of HCTZ or CTD and history of MI or stroke.

**Main Outcomes and Measures:**

Outcome ascertainment was performed from randomization to the end of the study. The primary outcome consisted of a composite of stroke, MI, urgent coronary revascularization because of unstable angina, acute heart failure hospitalization, or noncancer death. Additional outcomes included achieved blood pressure and hypokalemia (potassium level <3.1 mEq/L; to convert to mmol/L, multiply by 1.0).

**Results:**

The DCP randomized 13 523 participants to CTD or HCTZ, with a mean (SD) study duration of 2.4 (1.4) years. At baseline, median age was 72 years (IQR, 69-75 years), and 96.8% were male. Treatment effect was evaluated in subgroups of participants with (n = 1455) and without (n = 12 068) prior MI or stroke at baseline. There was a significant adjusted interaction between treatment group and history of MI or stroke. Participants with prior MI or stroke randomized to CTD had a lower risk of the primary outcome than those receiving HCTZ (105 of 733 [14.3%] vs 140 of 722 [19.4%]; hazard ratio [HR], 0.73; 95% CI, 0.57-0.94; *P* = .01) compared with participants without prior MI or stroke, among whom incidence of the primary outcome was slightly higher in the CTD arm compared with the HCTZ arm (597 of 6023 [9.9%] vs 535 of 6045 [8.9%]; HR, 1.12; 95% CI, 1.00-1.26; *P* = .054) (*P* = .01 for interaction). The incidence of a nadir potassium level less than 3.1 mEq/L and hospitalization for hypokalemia differed among those with and without prior MI or stroke when comparing those randomized to CTD vs HCTZ, with a difference only among those without prior MI or stroke (potassium level <3.1 mEq/L: prior MI or stroke, 43 of 733 [5.9%] vs 37 of 722 [5.1%] [*P* = .57]; no prior MI or stroke, 292 of 6023 [4.9%] vs 206 of 6045 [3.4%] [*P* < .001]; hospitalization for hypokalemia: prior MI or stroke, 14 of 733 [1.9%] vs 16 of 722 [2.2%] [*P* = .72]; no prior MI or stroke: 84 of 6023 [1.4%] vs 57 of 6045 [0.9%] [*P* = .02]).

**Conclusions and Relevance:**

Results of this secondary analysis of the DCP trial suggest that CTD may be associated with reduced major adverse CV events and noncancer deaths in patients with prior MI or stroke compared with HCTZ.

**Trial Registration:**

ClinicalTrials.gov Identifier: NCT02185417

## Key Points

**Question:**

Is there a difference in major adverse cardiovascular events (MACEs) and noncancer deaths associated with chlorthalidone (CTD) vs hydrochlorothiazide (HCTZ) among older patients with hypertension with and without prior myocardial infarction (MI) or stroke?

**Findings:**

In this secondary analysis of a randomized clinical trial including 13 523 patients aged 65 years or older, a significant qualitative interaction was found between treatment group and history of MI or stroke; participants with prior MI or stroke randomized to CTD had lower risk of MACEs and noncancer death than those receiving HCTZ. In participants without prior MI or stroke, outcomes were not significantly different.

**Meaning:**

Prior MI or stroke should be considered when deciding between CTD or HCTZ for the management of hypertension.

## Teaser

This secondary analysis of a randomized clinical trial investigates differences in incidence of cardiovascular outcomes and noncancer deaths in older patients receiving chlorthalidone vs hydrochlorothiazide who do and do not have a history of myocardial infarction or stroke.

## Introduction

Thiazide diuretics are commonly used to treat hypertension, as they both lower blood pressure (BP) and prevent cardiovascular (CV) events. Currently, they are recommended as first-line agents.^1^ There are mechanistic reasons that suggest chlorthalidone (CTD) may be superior to hydrochlorothiazide (HCTZ) for preventing CV outcomes. Chlorthalidone has a longer half-life and has been shown to be more effective in reducing 24-hour BP compared with HCTZ.^2^ Chlorthalidone also has other pleotropic effects, such as decreased platelet aggregation and vascular permeability mediated through inhibition of carbonic anhydrase.^3^ However, most thiazide prescriptions in the US are HCTZ rather than CTD,^4^ potentially because of adverse effects associated with CTD, such as hypokalemia.^5^

The Diuretic Comparison Project (DCP) was a pragmatic randomized clinical trial (RCT) comparing CTD with HCTZ for the treatment of hypertension to reduce major adverse CV events (MACEs) and noncancer deaths in older US veterans.^6^ A planned subgroup analysis involved comparing drug effects among participants with and without prior myocardial infarction (MI) or stroke to assess whether the effects differed when used for primary vs secondary CV prevention. The main trial results demonstrated no difference in effects between CTD and HCTZ on such outcomes when mostly low doses were used.^5^ However, there was a qualitative interaction between treatment group and baseline presence of MI or stroke. A qualitative interaction is when a treatment is beneficial in 1 subgroup but harmful in the other. The observed interaction was therefore unexpected and has not been described in prior studies, to our knowledge. We assessed whether treatment effects of CTD vs HCTZ associated with CV outcomes and adverse events were different in patients with prior MI or stroke and those without prior MI or stroke.

## Methods

### Study Design

The DCP was a multicenter, embedded, pragmatic, open-label RCT performed between June 2016 and June 2021 in the Veterans Affairs (VA) population (NCT02185417; trial protocol in Supplement 1). This prespecified secondary analysis was performed from January 3, 2023, to February 29, 2024. This study was approved by the VA central institutional review board with waivers of written informed consent documentation and Health Insurance Portability and Accountability Act authorization because there was minimal risk to participants. The trial design and results have been previously reported.^5^ Key eligibility criteria included (1) age of 65 years or older; (2) history of hypertension, with the last clinic systolic BP (SBP) measure of 120 mm Hg or greater; and (3) an active HCTZ prescription (25 or 50 mg daily). This study followed the Consolidated Standards of Reporting Trials (CONSORT) reporting guideline.^7^

Study intervention was embedded in each of the 72 participating VA health care systems. Primary care practitioners were identified and approached with electronic consent through the electronic health record (EHR) system. Once practitioners consented, their panel of patients was electronically screened to identify those meeting inclusion or exclusion criteria. Eligible participants were mailed study information and consented verbally by telephone. Patients were randomized to either continue HCTZ or switch to CTD at what were assumed to be pharmacologically comparable doses (eg, HCTZ, 25 mg daily, to CTD, 12.5 mg daily).

Study medications were filled as a usual care medication. After randomization, patients were considered to receive usual care, and all treatments and monitoring of hypertension were left to the primary care practitioners. The study did not define a BP target. The study required no additional clinical visits or data collection beyond usual care.

### Outcomes

The primary outcome was time from randomization to the first occurrence of a composite outcome consisting of MACE (stroke, MI, urgent coronary revascularization for unstable angina, or heart failure hospitalization) or noncancer death. Each component of the primary outcome was considered as an independent secondary outcome. Outcomes were evaluated by individuals blinded to treatment assignment. Outpatient visits were also ascertained. Drug fills from the VA pharmacy over the duration of the study were ascertained for all antihypertensive agents. This study specifically evaluated pharmacy fills for angiotensin converting enzyme (ACE) inhibitors; angiotensin receptor blockers (ARBs); mineralocorticoid receptor antagonists (MRAs), including spironolactone and eplerenone; loop diuretics; sodium-glucose cotransport protein 2 (SGLT2) inhibitors; and potassium supplementation.

### Expected Adverse Events

Both CTD and HCTZ are well-established treatments with known adverse events. Safety measures, including acute kidney injury and hypokalemia (potassium level <3.1 mEq/L; to convert to mmol/L, multiply by 1.0), were specified in the trial protocol (Supplement 1). Event monitoring was performed using VA EHRs and administrative claims.

### Data Collection and Outcome Ascertainment

Patient characteristics, including demographics, comorbidities, medication history, and BP, were extracted from EHRs and/or claims data. Race and ethnicity were included to ensure that our sample matched the population and so that subgroup analysis could be done to assess whether the outcomes were similar across race and ethnicity; categories were Black or African American (hereafter, *Black*), Hispanic or Latino, not Hispanic or Latino, White, other (included American Indian, Asian, Hawaiian, Pacific Islander, and multiracial), and unknown due to missing data. Study outcomes were ascertained using EHR data and national repositories. A list of predefined diagnosis and procedure codes using the *International Statistical Classification of Diseases and Related Health Problems, Tenth Revision* classification were applied to flag events of interest.^8^ Cardiovascular outcomes were ascertained using validated EHR phenotypes and confirmed using manual adjudication where needed.^9,10,11^ All death events were reviewed to determine whether they were related to cancer, which was later verified by the National Death Index.

### Statistical Analysis

A detailed statistical analysis plan for the DCP trial is provided in Supplement 1. The current analysis focused on assessing the treatment effect (CTD vs HCTZ) in a subgroup defined by the presence or absence of prior MI or stroke at baseline. The composite primary outcome was assessed using an unadjusted log-rank test stratified according to the participating VA health care systems. A Cox proportional hazards regression model, stratified according to the participating VA health care systems, was used to estimate the hazard ratios (HRs) for the primary outcome. The hazard functions were compared using a 2-sided log-rank test based on intention-to-treat principles. Secondary outcomes were assessed using Fine-Gray models^12^ to account for the competing risks with all-cause mortality. Adjusted models controlling for age, male sex, Black race, body mass index, history of diabetes, history of heart failure, smoking status, rurality of residence, and baseline use of SGLT2 inhibitors and/or MRAs are presented. The covariates were selected based on study protocol description and clinical relevance. Interaction between treatment assignment and the subgroup with MI or stroke was examined with multiplicity adjustment. The DCP protocol prespecified 7 subgroups to evaluate treatment effect among different strata.^9^ Therefore, the adjusted *P* value was computed for the 7 subgroups using the formula 1 − (1 − *P*)*^k^*, where *k* is the number of subgroups tested and *P* is the original unadjusted interaction *P* value.^13^

Baseline and follow-up measures were reported as mean (SD) or median (IQR) for continuous variables with nongaussian distributions by the Shapiro-Wilk test. Discrete or categorical variables were presented as frequencies (percentages). Overall changes in SBP and potassium levels were shown graphically with mean (SD). Unadjusted Fisher exact tests were used to assess group differences for categorical variables and Mann-Whitney *U* tests for continuous variables. Hazard ratios and the corresponding 95% CIs were reported. All analyses were performed using SAS software, version 9.4 (SAS Institute Inc) and followed the intent-to-treat principles. Two-sided *P* < .05 was considered statistically significant.

## Results

Overall, the DCP study, with a mean (SD) study duration of 2.4 (1.4) years, randomized 13 523 participants (eFigure 1 in Supplement 2). Median age was 72 years (IQR, 69-75 years); 96.8% were men, and 3.2% were women. Among the participants, 15.0% were Black; 3.7%, Hispanic or Latino; 92.8%, not Hispanic or Latino; 77.3%, White; 2.4%, other race and ethnicity; and 5.3%, with missing race and ethnicity data. A total of 1455 participants (10.8%) had a history of MI or stroke at baseline, whereas 12 068 (89.2%) did not. Compared with participants without prior MI or stroke, participants with prior MI or stroke were more likely to be Black, be current smokers, reside in urban areas, receive multiple antihypertensive drugs, have diabetes, and have an estimated glomerular filtration rate less than 60 mL/min/1.73m^2^ (eTable 1 in Supplement 2). Table 1 compares baseline characteristics by treatment and with stratification according to the presence of MI or stroke history at baseline; the baseline measurements were not different between treatment arms.

**Table 1.** Demographic and Baseline Information by Treatment and History of MI or Stroke vs No MI or Stroke^a^

Characteristic	Patients with prior MI or stroke (n = 1455)		Patients without prior MI or stroke (n = 12 068)	
	CTD (n = 733)	HCTZ (n = 722)	CTD (n = 6023)	HCTZ (n = 6045)
Age, median (IQR), y	72 (69-75)	72 (69-76)	71 (69-75)	71 (69-75)
Sex				
Female	17 (2.3)	14 (1.9)	203 (3.4)	197 (3.3)
Male	716 (97.7)	708 (98.1)	5820 (96.6)	5848 (96.7)
Race				
Black or African American	151 (20.6)	135 (18.7)	853 (14.2)	888 (14.7)
White	523 (71.4)	538 (74.5)	4706 (78.1)	4687 (77.5)
Other^b^	18 (1.2)	19 (2.6)	148 (2.5)	139 (2.3)
Unknown due to missing data	41 (5.6)	30 (4.2)	316 (5.3)	331 (5.5)
Ethnicity				
Hispanic or Latino	26 (3.5)	30 (4.2)	211 (3.5)	227 (3.8)
Not Hispanic or Latino	680 (92.8)	672 (93.1)	5601 (93.0)	5596 (92.6)
Resided in rural area^c^	306 (41.8)	309 (42.8)	2737 (45.4)	2770 (45.8)
BMI, median (IQR)	30 (27-34)	30 (27-34)	31 (28-35)	31 (28-35)
Medical history				
Diabetes	396 (54.0)	387 (53.6)	2571 (42.7)	2675 (44.3)
Heart failure	109 (14.9)	132 (18.3)	416 (6.9)	394 (6.5)
MI	230 (31.4)	258 (35.7)	NA	NA
Stroke	534 (72.9)	495 (68.6)	NA	NA
eGFR <60 mL/min/1.73m^2^	213 (30.8)	214 (31.0)	1337 (23.5)	1333 (23.4)
Current smoker	223 (32.2)	198 (29.4)	1297 (23.0)	1239 (22.0)
Systolic BP, median (IQR), mm Hg	136 (130-146)	137 (129-149)	136 (129-146)	136 (129-146)
Antihypertensive drugs used, median (IQR), No.	3 (2-4)	3 (2-4)	3 (2-3)	3 (2-3)
Hydrochlorothiazide used				
Received HCTZ, 25 mg	685 (93.5)	693 (96.0)	5694 (94.5)	5709 (94.4)
Alone	59 (8.1)	42 (5.8)	830 (13.8)	824 (13.6)
Plus 1 BP medication	191 (26.1)	201 (27.8)	2161 (35.9)	2083 (34.5)
Plus 2 BP medications	275 (37.5)	260 (36.0)	1905 (31.6)	1961 (32.4)
Plus 3 BP medications	172 (23.5)	162 (22.4)	889 (14.8)	928 (15.4)
Plus 4 BP medications	36 (4.9)	57 (7.9)	238 (4.0)	249 (4.1)
Other drugs used				
ACE inhibitor or ARB	496 (67.7)	509 (70.5)	3857 (64.0)	3887 (64.3)
Loop diuretic	20 (2.7)	26 (3.6)	119 (2.0)	146 (2.4)
SGLT2 inhibitor	29 (4.0)	34 (4.7)	144 (2.4)	140 (2.3)
MRA^d^	118 (16.1)	111 (15.4)	722 (12.0)	744 (12.3)

Abbreviations: ACE, angiotensin converting enzyme; ARB, angiotensin receptor blocker; BMI, body mass index (calculated as weight in kilograms divided by height in meters squared); BP, blood pressure; CTD, chlorthalidone; eGFR, estimated glomerular filtration rate; HCTZ, hydrochlorothiazide; MI, myocardial infarction; MRA, mineralocorticoid receptor antagonist; NA, not applicable; SGLT2, sodium-glucose cotransport protein 2.

^a^ Data are presented as number (percentage) of patients unless otherwise indicated.

^b^ Included American Indian, Asian, Hawaiian, Pacific Islander, and multiracial.

^c^ Based on the Veterans Affairs urban, rural, or highly rural classification.

^d^ Included spironolactone and eplerenone.

Over the trial duration, SBP was not different between treatments for both subgroups (Figure). Patients with prior MI or stroke had a greater median number of outpatient visits than those without a history of MI or stroke (12 [IQR, 7-23] vs 10 [IQR, 5-18]; *P* < .001). Median visit frequency did not differ by randomized group (CTD, 11 [IQR, 9-19] visits; HCTZ, 10 [IQR, 5-19] visits; *P* = .24). Those in the group with prior MI or stroke had similar use of an ACE inhibitor or ARB at year 2 compared with those without MI or stroke (51.5% vs 48.9%; *P* = .12) but were more likely to be using an SGLT2 inhibitor at 2 years (7.5% vs 3.9%; *P* < .001) (eTable 2 in Supplement 2).

**Figure.**
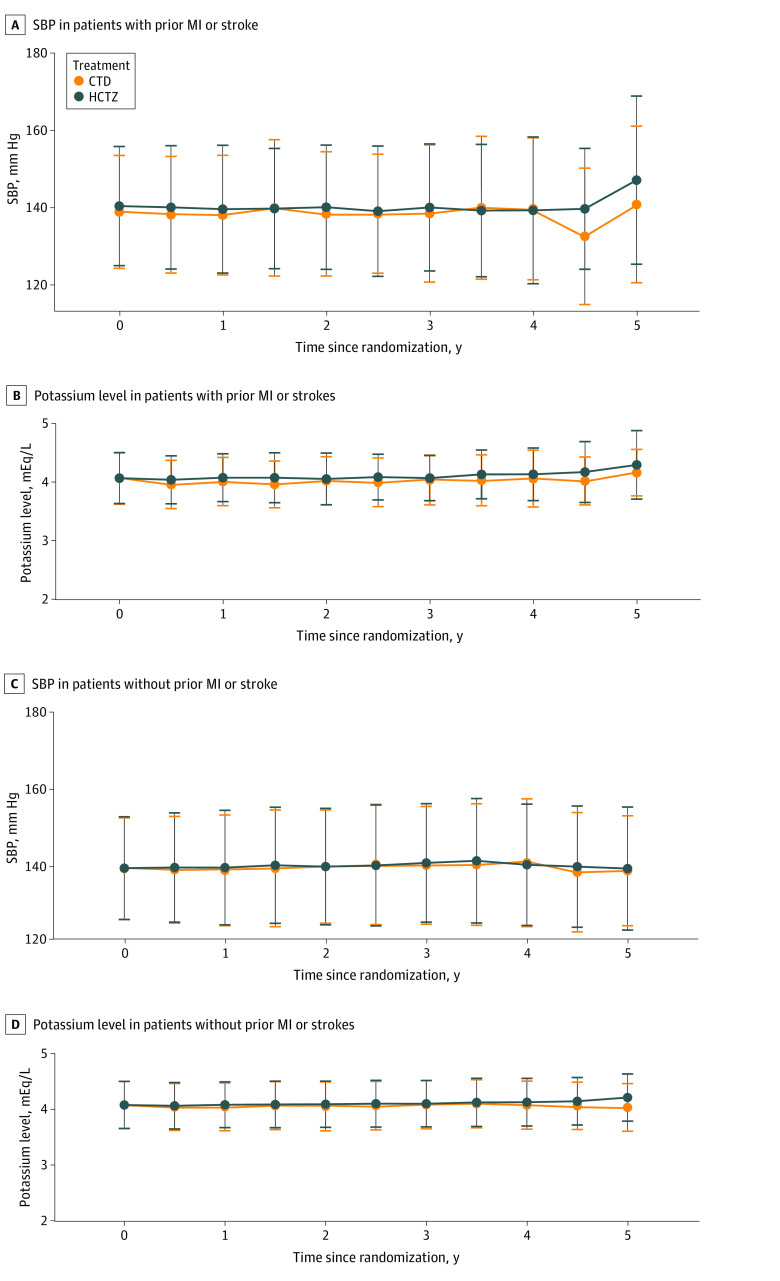
Systolic Blood Pressure (SBP) and Potassium Level Among Patients With and Without Prior Myocardial Infarction (MI) or Stroke Time 0 is the baseline. Whiskers indicate SDs. CTD indicates chlorthalidone; HCTZ, hydrochlorothiazide.

### Interaction

Analysis of the primary outcome demonstrated a significant qualitative interaction between treatment assignment and baseline history of MI or stroke (*P* = .002 for interaction). This interaction remained significant after adjustment for multiple comparisons (adjusted *P* = .01 for interaction). As shown in Table 2 and eFigure 2 in Supplement 2, in the group with prior MI or stroke, there was a lower risk of the primary outcome among those taking CTD vs HCTZ (105 of 733 [14.3%] vs 140 of 722 [19.4%]; HR, 0.73; 95% CI, 0.57-0.94; *P* = .01), resulting in an absolute risk reduction of 5.1% (95% CI, 4.0%-6.2%). The lower incidence of primary outcome events observed in the CTD arm vs the HCTZ arm was primarily attributable to a reduced risk of acute heart failure (34 of 733 [4.6%] vs 52 of 722 [7.2%]; HR, 0.64; 95% CI, 0.41-0.98). In contrast, those without prior MI or stroke had a slightly higher incidence of the primary outcome among those taking CTD compared with those taking HCTZ (597 of 6023 [9.9%] vs 535 of 6045 [8.9%]; HR, 1.12; 95% CI, 1.00-1.26; *P* = .054). Results did not change in the adjusted competing risk analysis (Table 2).

**Table 2.** Primary and Secondary Outcomes

Outcome	Patients, No./total No. (%)		Unadjusted analysis			Adjusted analysis^a^		
	Chlorthalidone	Hydrochlorothiazide	HR (95% CI)^b^	Log-rank *P* value	Interaction *P* value^c^	HR (95% CI)^b^	Log-rank *P* value	Interaction *P* value^c^
**Composite primary outcome**								
With MI or stroke history	105/733 (14.3)	140/722 (19.4)	0.73 (0.57-0.94)	.01	.002	0.74 (0.58-0.96)	.02	.004
Without MI or stroke history	597/6023 (9.9)	535/6045 (8.9)	1.12 (1.00-1.26)	.054		1.12 (1.00-1.26)	.054	
**Acute heart failure**								
With MI or stroke history	34/733 (4.6)	52/722 (7.2)	0.64 (0.41-0.98)	.04	.01	0.64 (0.42-1.00)	.054	.01
Without MI or stroke history	208/6023 (3.5)	180/6045 (3.0)	1.16 (0.95-1.42)	.15		1.17 (0.96-1.43)	.12	
**MI**								
With MI or stroke history	24/733 (3.3)	34/722 (4.7)	0.70 (0.42-1.18)	.18	.12	0.69 (0.40-1.16)	.16	.10
Without MI or stroke history	118/6023 (2.0)	106/6045 (1.8)	1.11 (0.86-1.45)	.42		1.13 (0.87-1.47)	.38	
**Stroke**								
With MI or stroke history	19/733 (2.6)	16/722 (2.2)	1.19 (0.62-2.32)	.60	.56	1.15 (0.59-2.24)	.68	.67
Without MI or stroke history	64/6023 (1.1)	67/6045 (1.1)	0.96 (0.68-1.35)	.79		0.98 (0.69-1.38)	.90	
**Unstable angina requiring revascularization**								
With MI or stroke history	4/733 (0.5)	3/722 (0.4)	1.34 (0.30-5.97)	.70	.83	1.48 (0.33-6.56)	.61	.91
Without MI or stroke history	16/6023 (0.3)	10/6045 (0.2)	1.60 (0.73-3.53)	.24		1.63 (0.74-3.60)	.23	
**Noncancer death**								
With MI or stroke history	55/733 (7.5)	76/722 (10.5)	0.72 (0.51-1.03)	.07	.04	0.75 (0.53-1.07)	.11	.06
Without MI or stroke history	304/6023 (5.0)	278/6045 (4.6)	1.09 (0.93-1.29)	.28		1.09 (0.93-1.29)	.29	

Abbreviations: HR, hazard ratio; MI, myocardial infarction.

^a^ Adjusted for age, male sex, Black race, body mass index, history of diabetes, history of heart failure, smoking status, rurality of residence, and baseline use of sodium-glucose cotransport protein 2 inhibitor and mineralocorticoid receptor antagonist. There were 88 participants with missing body mass index values at baseline.

^b^ Hazard ratios were estimated using a Cox proportional hazards regression model for the primary outcome and a Fine-Gray model for secondary outcomes to account for competing risk with all-cause mortality.

^c^ Interaction between study treatment and history of MI or stroke.

### Hypokalemia and Hypokalemic Adverse Events

There was a differential incidence of various degrees of hypokalemia by treatment group when stratified by history of MI or stroke. The incidence of a nadir potassium level between 3.1 and 3.5 mEq/L or less than 3.1 mEq/L was greater in the subgroup with prior MI or stroke than in the group without prior MI or stroke (Table 3). In the group with prior MI or stroke randomized to CTD, there was greater incidence of a nadir potassium level of 3.1 to 3.5 mEq/L compared with the HCTZ group (277 of 733 [37.8%] vs 206 of 722 [28.5%]; *P* < .001). However, the incidence of a nadir potassium level less than 3.1 mEq/L was similar between the CTD and HCTZ groups (43 of 733 [5.9%] vs 37 of 722 [5.1%]; *P* = .57). Participants receiving CTD were more likely to be prescribed potassium supplements at a median follow-up of 2.5 years (CTD, 76 of 401 [19.0%]; HCTZ, 52 of 414 [12.6%]; *P* = .01) (Table 4). In the subgroup with a history of MI or stroke, incidence of hospitalization for primary hypokalemia did not differ between the treatment groups (CTD, 14 of 733 [1.9%]; HCTZ, 16 of 722 [2.2%]; *P* = .72) (Table 3). In the subgroup with no prior MI or stroke randomized to CTD, there was similarly a greater incidence of a nadir potassium level of 3.1 to 3.5 mEq/L compared with those in the HCTZ group (1845 of 6023 [30.6%] vs 1592 of 6045 [26.3%]; *P* < .001) (Table 3), but there also was a greater incidence of a nadir potassium level less than 3.1 mEq/L in those randomized to CTD vs HCTZ (292 of 6023 [4.9%] vs 206 of 6045 [3.4%]; *P* < .001) (Table 3). Potassium supplement use was not different between treatments at a median follow-up of 2.5 years (CTD, 464 of 3463 [13.4%]; HCTZ, 417 of 3436 [12.1%]; *P* = .12) (Table 4). In addition, the frequency of hospitalizations for hypokalemia among those taking CTD was higher than that among those taking HCTZ in the subgroup without prior MI or stroke (84 of 6023 [1.4%] vs 57 of 6045 [0.9%]; *P* = .02) (Table 3).

**Table 3.** Adverse Events by Treatment and History of MI or Stroke vs No MI or Stroke

Adverse event	Patients with prior MI or stroke (n = 1455)			Patients without prior MI or stroke (n = 12 068)		
	Chlorthalidone (n = 733)	Hydrochlorothiazide (n = 722)	*P* value	Chlorthalidone (n = 6023)	Hydrochlorothiazide (n = 6045)	*P* value
**Serious adverse events**						
Any	280 (38.2)	293 (40.6)	.36	1710 (28.4)	1684 (27.9)	.52
All-cause mortality	65 (8.9)	88 (12.2)	.04	381 (6.3)	360 (6.0)	.40
Any hospitalization	260 (35.5)	271 (37.5)	.41	1565 (26.0)	1555 (25.7)	.76
**Expected adverse events**						
Any	166 (22.7)	172 (23.8)	.62	1231 (20.4)	1057 (17.5)	<.001
New allergic reaction to thiazide-type diuretic	5 (0.7)	4 (0.6)	>.99	104 (1.7)	17 (0.3)	<.001
Hospitalization for acute kidney injury	80 (10.9)	95 (13.2)	.20	415 (6.9)	417 (6.9)	>.99
Hyponatremia						
Any	52 (7.1)	53 (7.3)	.92	314 (5.2)	209 (5.1)	.81
Primary cause of hospitalization	25 (3.4)	22 (3.1)	.77	162 (2.7)	175 (2.9)	.51
Sodium level <130 mEq/L	37 (5.1)	36 (5.0)	>.99	191 (3.2)	160 (2.7)	.09
Hypokalemia						
Any	50 (6.8)	45 (6.2)	.67	356 (5.9)	253 (4.2)	<.001
Primary cause of hospitalization	14 (1.9)	16 (2.2)	.72	84 (1.4)	57 (0.9)	.02
Potassium level <3.1 mEq/L						
All	43 (5.9)	37 (5.1)	.57	292 (4.9)	206 (3.4)	<.001
Received potassium supplementation	25 (3.4)	25 (3.5)	>.99	203 (3.4)	137 (2.3)	<.001
Potassium level 3.1-3.5 mEq/L						
All	277 (37.8)	206 (28.5)	<.001	1845 (30.6)	1592 (26.3)	<.001
Received potassium supplementation	123 (16.8)	86 (11.9)	.01	732 (12.2)	621 (10.3)	.001

Abbreviation: MI, myocardial infarction.

SI conversion factors: To convert potassium and sodium level to millimoles per liter, multiply by 1.0.

**Table 4.** Potassium Supplementation by History of MI or Stroke vs No MI or Stroke

Follow-up period, y	Patients, No./total No. (%)	
	Chlorthalidone	Hydrochlorothiazide
With prior MI or stroke		
0.5	116/733 (15.8)	106/722 (14.7)
1	123/725 (17.0)	101/704 (14.3)
1.5	113/629 (18.0)	77/628 (12.3)
2	84/502 (16.7)	66/518 (12.7)
2.5	76/401 (19.0)	52/414 (12.6)
3	62/329 (18.8)	41/337 (12.2)
3.5	39/238 (16.4)	24/233 (10.3)
4	29/133 (21.8)	14/131 (10.7)
4.5	18/62 (29.0)	7/62 (11.3)
5	8/28 (28.6)	5/34 (14.7)
Without prior MI or stroke		
0.5	702/6023 (11.7)	669/6045 (11.1)
1	745/5973 (12.5)	722/5996 (12.0)
1.5	640/5334 (12.0)	625/5239 (11.9)
2	551/4395 (12.5)	514/4364 (11.8)
2.5	464/3463 (13.4)	417/3436 (12.1)
3	364/2840 (12.8)	330/2835 (11.6)
3.5	257/2019 (12.7)	225/2041 (11.0)
4	139/1155 (12.0)	128/1159 (11.0)
4.5	72/542 (13.3)	63/537 (11.7)
5	33/273 (12.1)	22/273 (8.1)

Abbreviation: MI, myocardial infarction.

### Adverse Events

In general, more adverse events occurred in those with prior MI or stroke compared with those without (573 of 1455 [39.4%] vs 3394 of 12 068 [28.1%]; *P* < .001), primarily due to a greater incidence of all-cause mortality (CTD, 65 of 733 [8.9%]; HCTZ, 88 of 722 [12.2%]; *P* = .04) and hospitalizations in those with a prior MI or stroke. There was no difference between treatments in the incidence of other, nonmortality-associated adverse events. Among those without prior MI or stroke, there was a greater risk of overall nonserious adverse events with CTD compared with HCTZ (1231 of 6023 [20.4%] vs 1057 of 6045 [17.5%]; *P* < .001), mostly attributable to hypokalemia (Table 3).

## Discussion

Overall findings of the DCP were reported previously; the HR for the primary outcome with CTD was 1.04 (95% CI, 0.94-1.16).^5^ In this prespecified secondary analysis of the DCP, there was a significant qualitative interaction between treatment assignment and the presence or absence of MI or stroke at baseline with respect to the primary outcome. In those with a prior MI or stroke, CTD appeared to be associated with lower incidence of MACE and noncancer-related deaths and with reduced all-cause mortality (not a prespecified outcome). An overall increase in incidence of hypokalemia in those randomized to CTD was noted irrespective of how hypokalemia was defined. The incidence of a nadir potassium level less than 3.1 mEq/L was not different between the CTD and HCTZ groups in those with prior MI or stroke. Among those without prior MI or stroke, there was a greater incidence of a nadir potassium level less than 3.1 mEq/L in the CTD group compared with the HCTZ group. This difference likely led to more hospitalizations for hypokalemia in the CTD group. It is unclear why, in the subgroup with prior MI or stroke, there was an increased incidence of a potassium level between 3.1 and 3.5 mEq/L in the CTD group compared with the HCTZ group but this did not translate to an increased incidence of a potassium level less than 3.1 mEq/L or an increased risk of hypokalemia-related hospitalizations. Of note, those taking CTD in the group with prior MI or stroke included a greater percentage of patients taking potassium supplements, which was not seen in those without prior MI or stroke. Use of medications moderating hypokalemia, such as ACE inhibitors, ARBs, or MRAs, while greater in those with a prior MI or stroke compared with those without prior MI or stroke, was not different in those randomized to CTD compared with HCTZ over the course of the study.

Hypokalemia has been associated with an increased risk of adverse CV outcomes.^14,15,16,17^ A meta-analysis of 13 cohort studies suggested a progressively increasing risk for mortality associated with hypokalemia starting at a potassium value of 3.5 mEq/L.^18^ Hypokalemia and its association with an increased risk for CV outcomes have been limiting factors for the use of high-dose diuretic therapy.^19,20^ A meta-analysis of 18 RCTs comparing high-dose vs low-dose diuretic therapy demonstrated a reduction in coronary heart disease and all-cause mortality associated with low-dose diuretics.^21^ Similarly, a secondary analysis of the Systolic Hypertension in the Elderly Program trial, which compared CTD with placebo in older individuals, demonstrated an increased risk of CV events in those taking CTD who developed hypokalemia (potassium level <3.5 mEq/L) compared with those who did not.^22^ The DCP trial was a pragmatic RCT with the assumption being that the doses of CTD and HCTZ were pharmacologically comparable. This was demonstrated by similar clinical BP values observed over the course of the trial. We also assumed that hypokalemia would be the same between groups and managed similarly, but this was not the case, as evidenced by the increased incidence of a nadir potassium level less than 3.1 mEq/L associated with CTD both overall in the DCP trial and specifically in participants without a prior MI or stroke. It is unclear why those with a prior MI or stroke had a greater incidence of mild hypokalemia but no greater incidence of moderate to severe hypokalemia or hospitalization for hypokalemia in the CTD group compared with the HCTZ group. Generally, patients were taking similar doses of HCTZ at baseline (6379 of 6756 [94.4%] and 6402 of 6767 [94.6%] were receiving 25 mg of HCTZ at baseline in the CTD and HCTZ group, respectively), and there was no difference in BP between treatment groups over the trial duration. Additionally, while those with prior MI or stroke were more likely to be taking an ACE inhibitor, ARB, and/or MRA at baseline, use of these classes of medications did not substantially change over the study course. On average, those in the group with prior MI or stroke had a greater number of outpatient visits over the duration of the study. The difference in use of potassium supplements without severe hypokalemia may have been the result of greater recognition of mild hypokalemia and greater treatment associated with the greater frequency of visits in this high-risk subgroup. However, the absolute number of moderate to severe hypokalemic events was greater in this group compared with the group without prior MI or stroke.

Other studies have demonstrated a similar increase in the incidence of hypokalemia associated with CTD compared with HCTZ.^23,24^ More recently, a study by Hripcsak et al^5^ using insurance databases suggested no difference in CV outcomes between CTD and HCTZ but an increased risk of hypokalemia associated with CTD. In addition, Edwards et al^25^ suggested in a propensity-matched cohort that CTD was associated with a greater risk for CV events and hypokalemia compared with HCTZ. It is unclear whether the overall results of the DCP trial would have been altered had hypokalemia (potassium level <3.5 mEq/L) been more aggressively monitored and treated. A hypothesis for the differential outcome in those with prior MI or stroke is that benefits of CTD were unmasked by the prevention of a nadir potassium level less than 3.1 mEq/L in the CTD compared with the HCTZ group and/or by the greater use of supplemental potassium in the CTD group.^26^ There may, however, be an alternative explanation.

Results of this analysis suggest a qualitative interaction. This type of interaction is less common than a quantitative interaction, in which the treatment is beneficial (or harmful) in all subgroups but the magnitude of effect varies among subgroups. It is typically difficult to interpret subgroups with qualitative interactions to determine whether the effect is real or simply from chance. Formal tests for interaction were conducted, with results remaining significant after adjusting for multiplicity. Also, the suggestion of a qualitative interaction by baseline MI or stroke status was seen for the primary outcome and for components of the primary outcome, including acute heart failure, MI, noncancer-related death, and total mortality. Finally, adverse effects related to hypokalemia differed in those with and without MI or stroke at baseline. Despite this approach, it is possible that the observed interaction was due to chance alone.

### Strengths and Limitations

Strengths of the current subgroup analysis include an a priori plan to examine the subgroup with MI or stroke and use of data prior to randomization. This study also has limitations. Despite the significant interaction in an a priori subgroup, these findings may be due to chance. While the incidence of adverse events was greater in the subgroup with prior MI or stroke than in the subgroup without MI or stroke, there were few participants in this group and there were fewer events overall. It is also unclear whether the benefits seen in the group with prior MI or stroke compared with the group with no prior MI or stroke were the result of baseline risk, prevention of hypokalemia, or additional potassium supplementation in the group with prior MI or stroke. We can only conclude that the combination of all 3 was associated with an improved outcome in participants taking CTD compared with HCTZ. There may be other factors not explored here that may have led to the observed interaction.

## Conclusions

The DCP study overall demonstrated no difference in outcomes between CTD and HCTZ when treating hypertension. This secondary analysis of the DCP trial found a significant qualitative interaction between treatment group and history of MI or stroke. The findings suggest that CTD may be associated with reduced MACEs and noncancer deaths in patients with prior MI or stroke compared with HCTZ. Accounting for this subgroup knowledge when treating patients, CTD may benefit those with prior MI or stroke. Future studies should evaluate this subgroup in further detail to determine whether these results are simply a chance finding or are real and potentially mediated through either hypokalemia prevention or potassium supplementation.

## Notes

Supplement 1.
Trial Protocol and Statistical Analysis Plan

Supplement 2. **eTable 1.** Demographic and Baseline Information by Prior MI or Stroke Status **eTable 2.** Use of Selected Medications Over Time **eFigure 1.** CONSORT Diagram by MI or Stroke Subgroup **eFigure 2.** Forest Plot by MI or Stroke Subgroup

Supplement 3.
Data Sharing Statement
