# Supplementary material for: Chlorthalidone vs Hydrochlorothiazide for Hypertension Treatment After Myocardial Infarction or Stroke: A Secondary Analysis of a Randomized Clinical Trial
Source: JAMA Netw Open. 2024 May 14;7(5):e2411081. doi: 10.1001/jamanetworkopen.2024.11081 (PMC11094558; doi:10.1001/jamanetworkopen.2024.11081)
Supplement: Supplement 2. — eTable 1. Demographic and Baseline Information by Prior MI or Stroke Status eTable 2. Use of Selected Medications Over Time eFigure 1. CONSORT Diagram by MI or Stroke Subgroup eFigure 2. Forest Plot by MI or Stroke Subgroup [file jamanetwopen-e2411081-s002.pdf]

Supplementary Online Content

Ishani A, Hau C, Cushman WC, et al. Chlorthalidone vs hydrochlorothiazide for hypertension treatment after myocardial infarction or stroke: a secondary analysis of a randomized clinical trial. *JAMA Netw Open*. 2024;7(5):e2411081. doi:10.1001/jamanetworkopen.2024.11081

eTable 1. Demographic and Baseline Information by Prior MI or Stroke Status

eTable 2. Use of Selected Medications Over Time

eFigure 1. CONSORT Diagram by MI or Stroke Subgroup

eFigure 2. Forest Plot by MI or Stroke Subgroup

This supplementary material has been provided by the authors to give readers additional information about their work.

© 2024 Ishani A et al. *JAMA Network Open*.

eTable 1. Demographic and Baseline Information by MI/stroke subgroup

	With prior MI/stroke (n=1,455)	Without prior MI/stroke (n=12,068)
Age, yrs - median (IQR)	72 (69 - 76)	71 (69 - 75)
Sex		
Female – n (%)	31 (3.3)	400 (3.3)
Male - n (%)	1,424 (97.9)	11,668 (96.7)
Race - n (%)		
Black or African American	286 (19.7)	1,741 (14.4)
White	1,061 (72.9)	9,393 (77.8)
Other[a]	37 (2.5)	287 (2.4)
Unknown due to missing data	71 (4.9)	647 (5.4)
Not Hispanic or Latino - n (%)	1,352 (92.9)	11,197 (92.8)
Resided in rural areas[b] - n (%)[a]	615 (42.3)	5,507 (45.6)
BMI, kg/m^2 – median (IQR)	30 (27 - 34)	31 (28 - 35)
Medical History - n (%)		
Diabetes	783 (53.8)	5,246 (43.5)
Heart failure	241 (16.6)	810 (6.7)
Myocardial infarction	488 (33.5)	---
Stroke	1,029 (70.7)	---
Estimated GFR <60 ml/min/1.73m^2	427 (30.9)	2,670 (23.5)
Current smoker - n (%)	421 (30.8)	2,536 (22.5)
HCTZ 25 mg - n (%)	1,378 (94.7)	11,403 (94.5)
Systolic blood pressure, mmHg – median (IQR)	136 (129 - 148)	136 (129 - 146)
Antihypertensive drug use, median (IQR)	3 (2 - 4)	3 (2 - 3)
Antihypertensive drug use – n (%)		
HCTZ alone	101 (6.9)	1,654 (13.7)
HCTZ plus 1 additional BP med	392 (26.9)	4,244 (35.2)
HCTZ plus 2 additional BP meds	535 (36.8)	3,866 (32.0)
HCTZ plus 3 additional BP meds	334 (23.0)	1,817 (15.1)
HCTZ plus 4 additional BP meds	93 (6.4)	487 (4.0)
Other drug use – n (%)		
ACE inhibitor or ARB	1,005 (69)	7,744 (64)
Loop diuretic	46 (3)	265 (2)
SGLT-2 inhibitor	63 (4)	284 (2)
MRA[c]	229 (16)	1,466 (12)

[a] Included Asian, Pacific islander, American Indian, Hawaiian, and respondents with multiple races.
[b] Based on the VA urban/rural/highly rural (URH) classification.
[c] Included spironolactone and eplerenone.

© 2024 Ishani A et al. *JAMA Network Open*.

eTable 2: Use of Selected Medications over Time

Prior Mi/stroke (n=1,455)	Baseline		6 months		12 months		18 months		24 months	
	CTD (n=733)	HCTZ (n=722)	CTD (n=725)	HCTZ (n=704)	CTD (N=629)	HCTZ (n=628)	CTD (n=502)	HCTZ (n=518)	CTD (n=401)	HCTZ (n=414)
ACEi	292 (40%)	307 (43%)	273 (38%)	294 (42%)	226 (36%)	255 (41%)	196 (39%)	208 (40%)	165 (41%)	151 (37%)
ARB	206 (28%)	204 (28%)	196 (27%)	201 (29%)	166 (26%)	179 (29%)	125 (25%)	145 (28%)	97 (24%)	115 (18%)
ACEi or ARB	496 (68%)	509 (71%)	466 (64%)	492 (70%)	391 (62%)	430 (69%)	318 (63%)	352 (68%)	260 (65%)	265 (64%)
SGLT2i	29 (4%)	34 (5%)	38 (5%)	43 (6%)	43 (7%)	57 (9%)	38 (8%)	44 (9%)	38 (10%)	38 (9%)
MRA	118 (16%)	111 (15%)	133 (18%)	120 (17%)	124 (20%)	107 (17%)	105 (21%)	89 (17%)	84 (21%)	77 (19%)
Loop diuretics	20 (2.7%)	26 (3.6%)	28 (3.9%)	42 (6.0%)	33 (5.2%)	51 (8.1%)	34 (6.8%)	49 (9.5%)	37 (9.2%)	52 (12.6%)
**No prior Mi/stroke (n=12,068)**	**Baseline**		**6 months**		**12 months**		**18 months**		**24 months**	
	CTD (n=6,023)	HCTZ (n=6,045)	CTD (n=5,973)	HCTZ (n=5,996)	CTD (N=5,334)	HCTZ (n=5,329)	CTD (n=4,395)	HCTZ (n=4,364)	CTD (n=3,463)	HCTZ (n=3,435)
ACEi	2,183 (36%)	2,226 (37%)	2,111 (35%)	2,173 (36%)	1,875 (35%)	1,905 (36%)	1,493 (34%)	1,555 (36%)	1,152 (33%)	1,197 (35%)
ARB	1,692 (28%)	1,685 (28%)	1,679 (28%)	1,703 (29%)	1,509 (28%)	1,493 (29%)	1,269 (29%)	1,217 (28%)	1,008 (29%)	948 (28%)
ACEi or ARB	3,857 (64%)	3,887 (64%)	3,772 (63%)	3,856 (64%)	3,364 (63%)	3,390 (64%)	2,740 (62%)	2,760 (63%)	2,150 (62%)	2,133 (62%)
SGLT2i	144 (2%)	140 (2%)	217 (4%)	220 (4%)	226 (4%)	211 (4%)	199 (5%)	189 (4%)	171 (5%)	169 (5%)
MRA	722 (12%)	744 (12%)	792 (13%)	794 (13%)	783 (15%)	745 (14%)	643 (15%)	645 (15%)	545 (16%)	526 (15%)
Loop diuretics	119 (2.0%)	146 (2.4%)	168 (2.8%)	214 (3.6%)	220 (4.1%)	251 (4.7%)	212 (4.8%)	266 (6.1%)	199 (5.7%)	235 (6.8%)

© 2024 Ishani A et al. *JAMA Network Open*.

eFigure 1. CONSORT diagram by MI/stroke subgroup

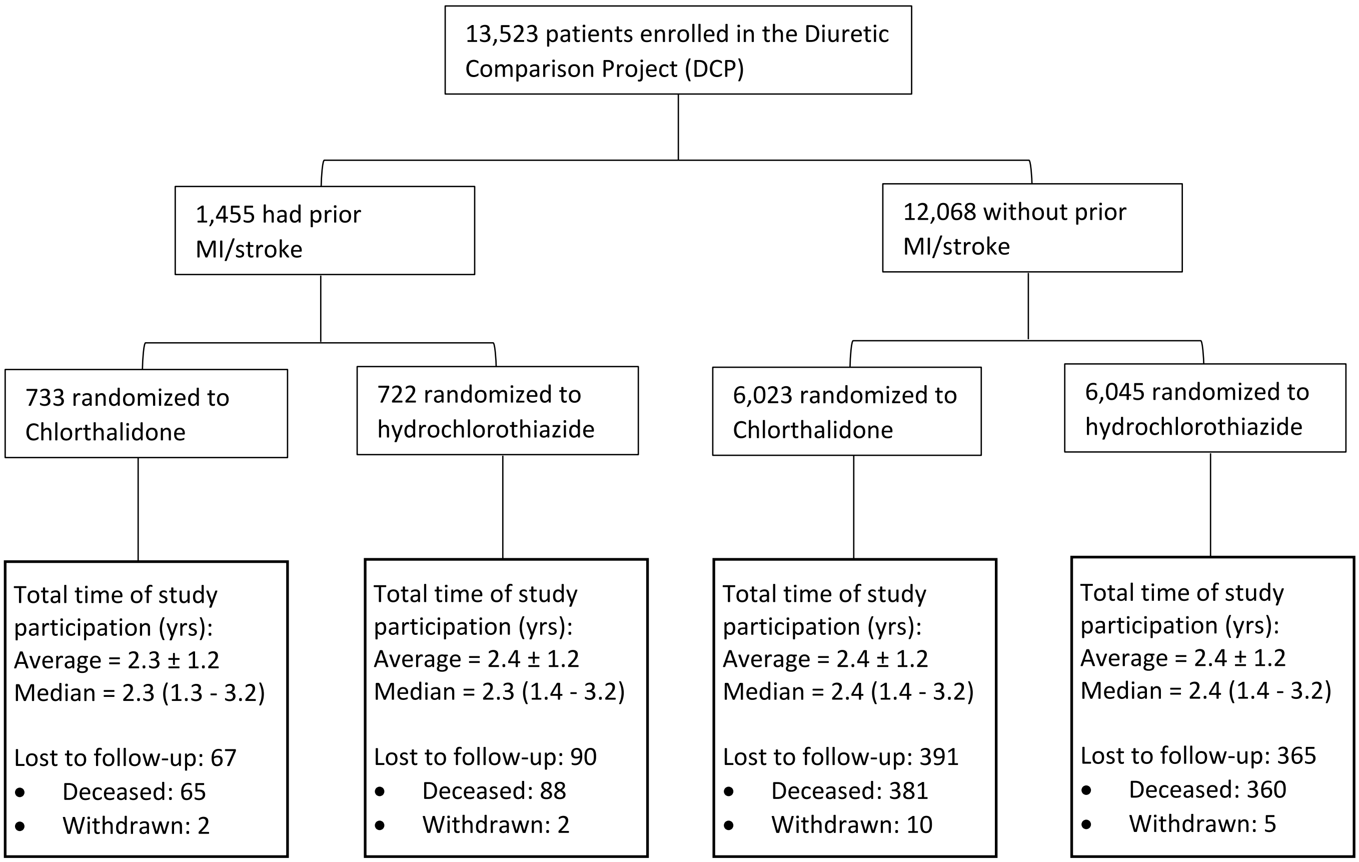

© 2024 Ishani A et al. *JAMA Network Open*.

eFigure 2. Forest plot by MI/stroke subgroup

Study Outcomes	Percentage of patients	HR (95% CI)
Patients with prior MI/stroke (n=1,455)		
Composite primary outcome	CTD (14.3%) / HCTZ (19.4%)	0.73 (0.57 - 0.94)
Acute heart failure	CTD (4.6%) / HCTZ (7.2%)	0.64 (0.41 - 0.98)
Myocardial infarction	CTD (3.3%) / HCTZ (4.7%)	0.70 (0.42 - 1.18)
Stroke	CTD (2.6%) / HCTZ (2.2%)	1.19 (0.62 - 2.32)
Unstable angina requiring revascularization	CTD (0.5%) / HCTZ (0.4%)	1.34 (0.30 - 5.97)
Non-cancer death	CTD (7.5%) / HCTZ (10.5%)	0.72 (0.51 - 1.03)
Patients without prior MI/stroke (n=12,068)		
Composite primary outcome	CTD (9.9%) / HCTZ (8.9%)	1.12 (1.00 - 1.26)
Acute heart failure	CTD (3.5%) / HCTZ (3.0%)	1.16 (0.95 - 1.42)
Myocardial infarction	CTD (2.0%) / HCTZ (1.8%)	1.11 (0.86 - 1.45)
Stroke	CTD (1.1%) / HCTZ (1.1%)	0.96 (0.68 - 1.35)
Unstable angina requiring revascularization	CTD (0.3%) / HCTZ (0.2%)	1.60 (0.73 - 3.53)
Non-cancer death	CTD (5.0%) / HCTZ (4.6%)	1.09 (0.93 - 1.29)

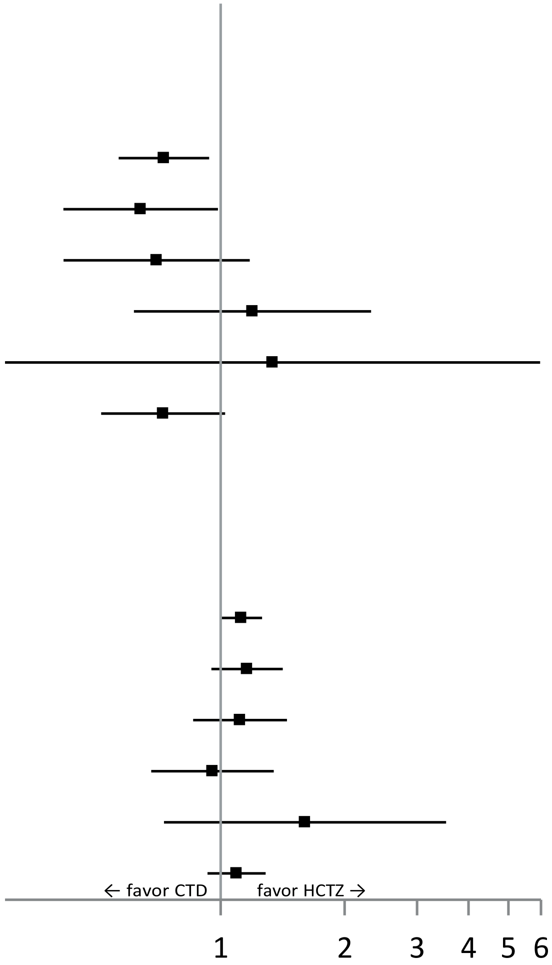

Hazard ratios are shown on a logarithmic scale

© 2024 Ishani A et al. *JAMA Network Open*.
