# Supplementary material for: Chlorthalidone vs Hydrochlorothiazide for Hypertension Treatment After Myocardial Infarction or Stroke: A Secondary Analysis of a Randomized Clinical Trial
Source: JAMA Netw Open. 2024 May 14;7(5):e2411081. doi: 10.1001/jamanetworkopen.2024.11081 (PMC11094558; doi:10.1001/jamanetworkopen.2024.11081)
Supplement: Supplement 3. — Data Sharing Statement [file jamanetwopen-e2411081-s003.pdf]

Data Sharing Statement

Ishani. Chlorthalidone vs Hydrochlorothiazide for Hypertension Treatment After Myocardial Infarction or Stroke. *JAMA Netw Open*. Published May 14, 2024.
doi:10.1001/jamanetworkopen.2024.11081

Data
Data available: No

Additional Information
Explanation for why data not available: The datasets used or generated from the current study are not publicly available. De-identified, aggregated data may be provided upon request through an approved VA Data Use Agreement.
